# Clinical Efficacy of a 2-Week Treatment Course of Zuranolone for the Treatment of Major Depressive Disorder and Postpartum Depression: Outcomes From the Clinical Development Program

**DOI:** 10.1192/j.eurpsy.2022.282

**Published:** 2022-09-01

**Authors:** A. Clayton, A.J. Cutler, K.M. Deligiannidis, R. Lasser, A.J. Sankoh, J. Doherty, M. Kotecha

**Affiliations:** 1 University of Virginia School of Medicine, Department Of Psychiatry And Neurobehavioral Sciences, Charlottesville, United States of America; 2 SUNY Upstate Medical University, Department Of Psychiatry, Syracuse, United States of America; 3 Zucker Hillside Hospital, Department Of Psychiatry, Glen Oaks, United States of America; 4 Sage Therapeutics, Inc, Clinical Development, Cambridge, United States of America; 5 Sage Therapeutics, Inc, Data Science, Cambridge, United States of America; 6 Biogen, Clinical Development, Cambridge, United States of America

**Keywords:** postpartum depression, zuranolone, rapid onset of action, major depressive disorder

## Abstract

**Introduction:**

Antidepressants that offer a rapid onset of action without requiring chronic use are greatly needed in both major depressive disorder (MDD) and postpartum depression (PPD). Zuranolone is an investigational, oral, neuroactive steroid and GABA_A_ receptor positive allosteric modulator in clinical development as a 2-week treatment course for MDD and PPD.

**Objectives:**

To present the efficacy and safety of zuranolone vs placebo in Phase 2 and 3 trials.

**Methods:**

In the studies presented (**Table 1**), improvements in depressive symptoms were assessed by least-squares mean (LSM) using a mixed-effects model for repeated measures on the change from baseline (CFB) at Day 15 in the 17-item Hamilton Rating Scale for Depression total score (HAMD-17; primary endpoint for all trials) and the Montgomery–Åsberg Depression Rating Scale (MADRS; secondary endpoint) following a 14-day treatment course of once-daily zuranolone.

**Figure:**
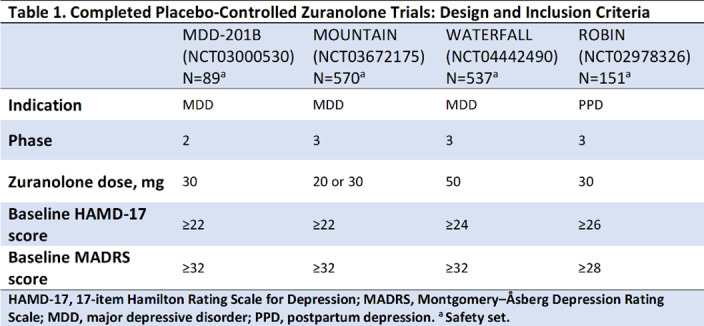

**Results:**

Compared with placebo, zuranolone treatment led to rapid improvements in depressive symptoms across clinical trials, with significant improvements (LSM treatment difference [SE] in CFB) in HAMD-17 and MADRS scores at Day 15 in 3 of the 4 trials (**Table 2**). Common treatment-emergent adverse events (≥5% in zuranolone treatment arms) were headache, somnolence, dizziness, nausea, sedation, diarrhea, upper respiratory tract infection, and fatigue (**Table 3**). No incidences of loss of consciousness or excessive sedation were observed.

**Figure:**
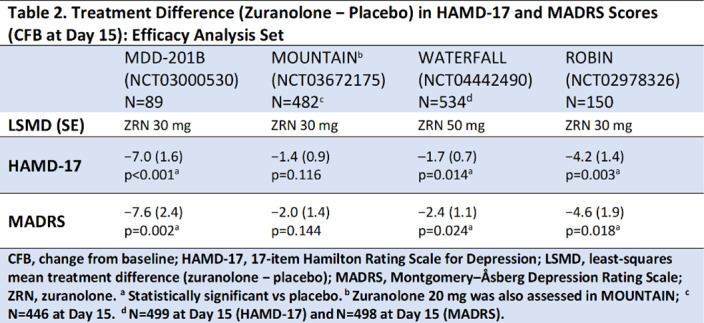

**Figure:**
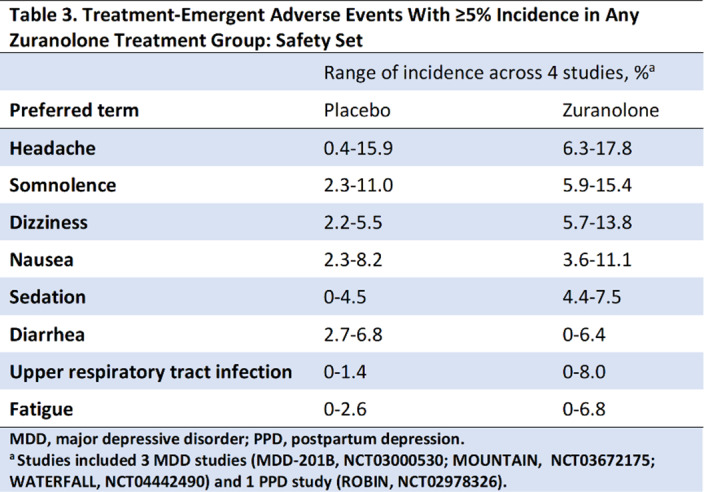

**Conclusions:**

Across the completed studies in the zuranolone clinical trial program, patients receiving zuranolone consistently experienced improvement in depressive symptoms following a 2-week treatment course. Treatment with zuranolone was generally well tolerated with a consistent safety and tolerability profile.

**Disclosure:**

The MDD-201B, MOUNTAIN, and ROBIN studies were sponsored by Sage Therapeutics, Inc; the WATERFALL study was sponsored by Sage Therapeutics, Inc, and Biogen. Medical writing and editorial support were provided by MediTech Media, Ltd, and funded by Biogen.

